# Polydopamine-assisted aptamer-carrying tetrahedral DNA microelectrode sensor for ultrasensitive electrochemical detection of exosomes

**DOI:** 10.1186/s12951-024-02318-6

**Published:** 2024-02-08

**Authors:** Bowen Jiang, Tenghua Zhang, Silan Liu, Yan Sheng, Jiaming Hu

**Affiliations:** 1 International Joint Laboratory of Catalytic Chemistry, State Key Laboratory of Advanced Special Steel, Innovation Institute of Carbon Neutrality, College of Sciences, Shanghai University, Shanghai, 200444 China; 2 Institute of Translational Medicine, Shanghai University, Shanghai, 200444 China; 3 MOE Key Laboratory of Laser Life Science & Institute of Laser Life Science, Guangdong Provincial Key Laboratory of Laser Life Science, College of Biophotonics, South China Normal University, Guangzhou, 510631 China

**Keywords:** Exosomes, Aptamer, Polydopamine, Tetrahedra DNA, Microelectrode, Electrochemical sensing

## Abstract

**Background:**

Exosomes are nanoscale extracellular vesicles (30–160 nm) with endosome origin secreted by almost all types of cells, which are considered to be messengers of intercellular communication. Cancerous exosomes serve as a rich source of biomarkers for monitoring changes in cancer-related physiological status, because they carry a large number of biological macromolecules derived from parental tumors. The ultrasensitive quantification of trace amounts of cancerous exosomes is highly valuable for non-invasive early cancer diagnosis, yet it remains challenging. Herein, we developed an aptamer-carrying tetrahedral DNA (Apt-TDNA) microelectrode sensor, assisted by a polydopamine (PDA) coating with semiconducting properties, for the ultrasensitive electrochemical detection of cancer-derived exosomes.

**Results:**

The stable rigid structure and orientation of Apt-TDNA ensured efficient capture of suspended exosomes. Without PDA coating signal amplification strategy, the sensor has a linear working range of 10^2^–10^7^ particles mL^−1^, with LOD of ~ 69 exosomes and ~ 42 exosomes for EIS and DPV, respectively. With PDA coating, the electrochemical signal of the microelectrode is further amplified, achieving single particle level sensitivity (~ 14 exosomes by EIS and ~ 6 exosomes by DPV).

**Conclusions:**

The proposed PDA-assisted Apt-TDNA microelectrode sensor, which integrates efficient exosome capture, sensitive electrochemical signal feedback with PDA coating signal amplification, provides a new avenue for the development of simple and sensitive electrochemical sensing techniques in non-invasive cancer diagnosis and monitoring treatment response.

**Graphical Abstract:**

**Figure:**
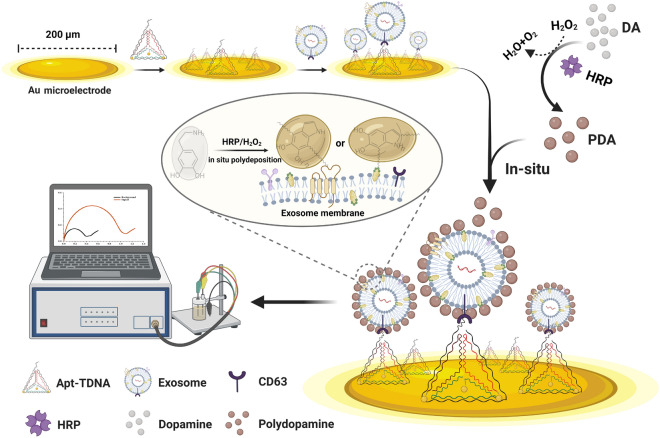

**Supplementary Information:**

The online version contains supplementary material available at 10.1186/s12951-024-02318-6.

## Introduction

Exosomes, nanoscale endocrine vesicles (30–160 nm), are considered as promising biomarkers for early-stage cancer diagnosis. They are ubiquitous and stable in almost all biological fluids [1] and are considered as messengers to mediate intercellular communication [2]. Discovered by tracing the origin of tumor tissue, in the early stage of tumorigenesis, tumor cells continuously secrete exosomes that carry tumor molecular tags such as oncoproteins, microRNAs, and DNAs into the bloodstream to assist in the transformation and phenotypic reprogramming of recipient cells, thereby promoting tumor formation [3–5]. The diverse cargo within exosomes not only indicates their cellular origin but also serves as an important indicator of cancer-related physiological changes [6–9].

Direct observation and detection of exosomes pose significant challenges due to their high heterogeneity and nanometer size [10]. The low concentrations of tumor cells and their derived exosomes in the early stage of tumorigenesis further emphasize the sensitivity of detection methods. Current research on exosome detection mainly focuses on surface plasmon resonance-based [11, 12], surface-enhanced Raman spectroscopy-based [12], fluorescence signal amplification-based [13–15], microfluidic platform-dependent [16, 17] and electrochemical biosensing [18–23]. Among these, electrochemistry stands out in biosensing due to its superior sensitivity, rapid response, small sample volume, and scalability. However, existing studies on the electrochemical detection of exosomes often suffer from complexity, time consumption, and dependence on enzyme tools such as Exo III and CRISPR/Cas [20, 21, 24–26]. Hence, it is desirable to develop a simple, fast, accurate, and cost-effective electrochemical technology for exosome detection.

**Fig. 1:**
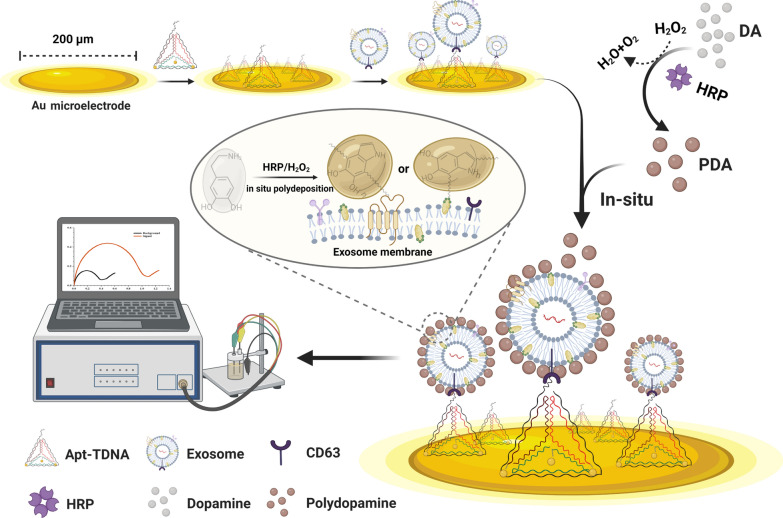
Schematic illustration of the PDA coating assisted Apt-TDNA microelectrode sensor

Here, we report a polydopamine (PDA)-assisted aptamer-carrying tetrahedral DNA (Apt-TDNA) microelectrode sensor for electrochemical detection of exosomes (Fig. 1). The sensor targets CD63 protein on the membrane of A549 cell-derived exosomes [27, 28] by Apt-TDNA. Then, monomer dopamine (DA) is catalyzed by horseradish peroxidase (HRP) to form PDA in the presence of hydrogen peroxide [29]. PDA [30, 31], a mussel bionic material, settles on the exosome membrane surface by Michael addition or Schiff base reaction of catechol with amino and thiol groups [29, 30, 32, 33], effectively hindering charged particles from reaching the electrode surface. The resulting differential electrical signal can be monitored by electrochemical impedance spectroscopy (EIS) and differential pulse voltammetry (DPV) of the electrochemical workstation.

In this work, instead of using traditional 4 mm and screen-printed electrodes, we chose a gold disk microelectrode with a reduced working area of 0.2 mm in diameter. Microelectrodes exhibit multiple unique electrochemical properties [34, 35]. First, the small resistance–capacitance (RC) time constant of the microelectrode enables excellent performance in fast and transient steady-state electrochemical reactions. Additionally, microelectrodes benefit from high-density working currents and exhibit minimal polarization currents, mitigating the IR (voltage) drop within the system, which is suitable for establishing high-impedance systems. Moreover, the exceptionally rapid material diffusion rate on the microelectrode surface facilitates a clear distinction from charge transfer diffusion, thereby enhancing the detection process of EIS.

Aptamers, often referred to ‘chemical antibodies’, are artificially synthesized short oligonucleotides that exhibit highly specific affinity and selectivity toward their target molecules [36, 37]. Compared with single-stranded nucleic acid aptamers, Apt-TDNAs are ordered and uniformly arranged on the electrode surface, effectively mitigating entanglement and aggregation commonly associated with single-stranded nucleic acid aptamers [38–41], thereby improving the binding efficiency to exosomes.

## Experimental design

### Reagents

All DNA oligonucleotides (Additional file 1: Table S1), with or without thiol labeling, were designed using the NUPACK to prevent unintended secondary structures. These oligonucleotides were synthesized and purified by Sangon Biotechnology Co., Ltd. (Shanghai, China). Dopamine (DA) was purchased from Aladdin Reagent (Los Angeles, Southern California, USA). 6-mercaptohexanol, horseradish peroxidase (HRP), tris 2-carboxyethyl phosphine hydrochloride (TCEP), 1-(3-dimethylaminopropyl)-3-ethylcarbodiimide hydrochloride (EDC), BSA, DiO dye, Tween20, Tris–HCl buffer (pH 8.0), PBS buffer (pH 7.4) and TE buffer were purchased from Shanghai Shenggong Biotechnology Co., Ltd (Shanghai, China). Sulfuric acid (H_2_SO_4_), hydrogen peroxide (H_2_O_2_), potassium ferricyanide (K_3_[Fe(CN)_6_]) and potassium ferrocyanide (K_4_[Fe(CN)_6_]) were purchased from Guangzhou Chemical Reagent Co., Ltd (Guangzhou, China). RPMI-1640 cell culture medium, fetal bovine serum (FBS) and penicillin/streptomycin were purchased from Gibco (Grand Island, New York, USA). APTES was purchased from Sigma-Aldrich (St. Louis, Mississippi, USA). Gold microelectrodes with a diameter of 0.2 μm were purchased from Shanghai Xianren Instrument Co., Ltd (Shanghai, China). Pt counter electrodes and Ag/AgCl reference electrodes were purchased from Gao Shi Rui Lian Optoelectronic Technology Co., Ltd. Deionized (DI) water (Millipore Milli-Q grade, 18.2 MΩ) was used throughout the experiments.

### Equipment

The 5417R high-speed refrigerated centrifuge produced by Eppendorf and the Optima MAX-XP refrigerated ultracentrifuge produced by Beckman Coulter were used to isolate and purify exosomes from the supernatant of cell culture. The nanoparticle tracking analysis (Nanosight SN300, Malvern) was used for quantifying exosomes. The JEM-1400 PLUS transmission electron microscope produced by Japan Electronics Co., Ltd. was used to observe the morphology of exosomes. The Cypher ES atomic force microscope (AFM) produced by Oxford Instruments was used to observe the morphology of Apt-TDNA. CytoFLEX flow cytometer produced by Beckman Coulter was used to characterize CD63 membrane protein of exosome. The CHI 660e electrochemical workstation produced by CH Instruments was used for electrochemical detection of exosomes.

### Cell culture and exosome isolation

A549 (National Collection of Authenticated Cell Cultures, Shanghai, China) is a human lung cancer cell line, which was chosen as the test cell. All cell lines have been tested for mycoplasma contamination. Cells were grown in T25 cell culture flasks and expanded to 8 flasks for the collection of exosomes. Cells were grown and passaged in RPMI-1640 (10% FBS, 1% penicillin/streptomycin) and incubated at 37 ℃ with 5% CO_2_. For the starvation of cells, once the cells are grown to about 80% confluence, the media was changed to 5 mL of RPMI-1640 (without FBS, 1% penicillin/streptomycin), and the supernatant was collected after 40 h. 40 mL of cell supernatant was collected and spun at 300 g for 10 min, 2000 g for 10 min, and 10000 g for 30 min at 4 ℃ to remove cells and cell debris. Then, the supernatant was filtered through a 0.22 μm pore filter and followed by ultracentrifugation at 100,000 × g for 75 min at 4 ℃ to retain the pellet of exosomes. The pellet of exosomes was resuspended with 10 mL of PBS and precipitated by the second ultracentrifugation at 100,000 × g for 75 min at 4 ℃. The pellet of exosomes was resuspended in 800 μL of PBS and stored at − 80 ℃ for later use. The concentration of exosomes used in this method is 5.05 × 10^9^ particles mL^−1^, which is derived from the mean value of three independent tests of nanoparticle tracking analysis (NTA).

### Characterization of CD63 membrane protein of exosome

The carboxyl magnetic beads (Bangs Laboratories, Indiana, USA) were first functionalized with amino-modified aptamers. To achieve this, 5 µL of magnetic bead stock solution was mixed with 200 µL of 0.1 M MES buffer and placed on a magnetic stand (CISTRO, Guangzhou, China) for 5 min, after which the supernatant was discarded. Next, 50 µL of MES buffer, 20 µL of 10 µM CD63 aminated aptamer, and 10 µL of EDC buffer were added. The mixture was continuously mixed for 20 min, with an additional 10 µL of EDC buffer added twice during this process. After the last addition of EDC buffer, the mixture was further mixed for 100 min. Subsequently, 200 µL of 0.02% Tween/PBS was added for two rounds of washing. Following this, 200 µL of 1 M ethanolamine was added and incubated for 30 min. After two washes with 200 µL of TE buffer, the beads were resuspended in 50 µL of TE buffer and stored at 4 °C for later use. For staining, 5 µL of 250 µM DiO dye was added to 20 µL of fresh exosomes, which were then water-bathed at 37 °C for 30 min. Simultaneously, 2 µL of aptamer-functionalized magnetic beads were washed with PBS and blocked with 25 µL of 0.5% BSA/PBS with 550 nM Mg^2+^ to prevent non-specific adsorption. The stained exosomes were mixed with the blocked magnetic beads and incubated at 37 °C for 30 min, with intermittent mixing for 10 min and magnetic absorption for 5 min. The supernatant was discarded after magnetic absorption, and the beads were washed with PBS. Finally, 100 µL of PBS was added for detection using a CytoFLEX flow cytometer. Bare magnetic beads were treated identically as controls.

### Preparation of tetrahedra DNA

First, 1 µM of each of the four strands of the tetrahedron was treated with 3 mM freshly prepared TCEP in 1.5 × PBS for 2 h at room temperature in the dark to cleave the disulfide linkage. Subsequently, the mixture was then heated to 95 ℃ for 10 min in polymerase chain reaction (PCR) instrument, and rapidly cooled in an ice-water mixture to form a stable tetrahedral structure. The Ram-DNA with THa2, THb, THc, and THd was prepared using the same method. The formation of DNA nanostructures was analyzed using 2.5% agarose gel electrophoresis. Prepared Apt-TDNA can be stored at 4 ℃ for at least 1 week.

### Characterization of Apt-TDNA using AFM

Initially, 40 µL of a freshly prepared 0.5% (wt/vol) APTES anhydrous ethanol solution was added to the center of the newly uncovered mica sheet surface and incubated for 2 min. Subsequently, the surface was rinsed with a large amount of deionized water to remove excess APTES and dried using nitrogen. Then, 20 µL of freshly prepared 100 nM Apt-TDNA was added to the center of the treated mica sheet. After a 15 min incubation, 80 µL of TM buffer (50 mM Tris HCl, 10 mM magnesium sulfate, pH 7.5) was added. Scanning imaging was performed using the liquid phase tapping mode of the Cypher ES AFM at a scanning speed of 2.44 Hz, with the BLAC-40 probe being used.

### Pretreatment of gold microelectrode

Initially, the gold microelectrode was immersed in fresh piranha solution (H_2_SO_4_/H_2_O_2_, 7: 3) for 5 min and rinsed with deionized water. (Caution: piranha solution is highly corrosive and should be handled with extreme care, in small amounts only). Next, it was ultrasonically cleaned in ethanol and deionized water for 30 s each. Then, the gold microelectrode was meticulously polished to achieve a mirror surface using a polishing pad with evenly distributed 0.01 µm diamond particles (Mipox, Tokyo, Japan), coupled with a polishing compound containing 75 nm silicon dioxide particles. Following a wash with deionized water, the microelectrode was once again ultrasonically cleaned in ethanol and deionized water for 30 s, repeating this step. Subsequently, the microelectrode was dried using a nitrogen gas stream. The morphology of microelectrode was observed at 400-fold magnification with an optical microscope, exhibiting a mirror-like appearance without evident scratches (Additional file 1: Fig. S2b). Finally, the treated microelectrode was electrochemically polished by performing cyclic voltammetry [42] in a 0.5 M sulfuric acid solution until the anodic characteristic peak current of gold stabilized (Additional file 1: Fig. S3a). The microelectrode was then scanned using CV in steps of 50 mV in an electrolyte buffer (5 mM [Fe (CN)_6_]^3−/4−^, 0.1 M KCl) (Additional file 1: Fig. S3b). The potential difference between the oxidation peak (Ep)a and the reduction peak (Ep)c ranged from 65 to 90 mV, demonstrating the effective cleaning of the microelectrode surface. The closer the peak currents of the anode (ip)a and the cathode (ip)c are, the greater the electrochemical reversibility of the microelectrode surface. Such microelectrode is suitable for subsequent assembly.

### Electrochemical detection protocol

The cleaned microelectrode was immersed in freshly prepared Apt-TDNA, sealed with parafilm, and incubated at room temperature in the dark for 2 h. (Note: the sensor can be stored in a bottle with N_2_ gas at 4 ℃, and the bottom of the bottle contains a small amount of deionized water to ensure humidity). Following this, the interface was washed with PBS and then immersed in a 1 mM MCH for 30 min [43]. Subsequently, an equal volume of the ‘age’ solution (1 × PBS, 1 M NaCl, pH 7.4) was added, and the solution was allowed to incubate under these conditions for an additional 2 h. Unbound reagents were thoroughly removed by washing with deionized water. Next, 100 µL of exosomes were incubated with the Apt-TDNA microelectrode for 90 min at room temperature. The electrodes were then washed with an electrolyte buffer, and each step was characterized using EIS with an amplitude of 10 mV and a frequency range of 0.1–10^5^ Hz, as well as DPV with an amplitude of 50 mV and a step size of 2 mV.

### PDA coating

The DA solution was prepared by dissolving 50 mg of DA in 10 mL of 10 mM Tris–HCl (pH 8.0). For every 100 µL of the DA solution, 20 µL of 3% H_2_O_2_ and 15 µL of 900 nM HRP were added. The mixture was incubated at room temperature for 1 h, allowing the colorless DA to oxidize and polymerize into brown PDA. After capturing EVs, the Apt-TDNA microelectrode was immersed in the PDA solution for 90 min to create a coating. The electrode was then washed with an electrolyte buffer. To eliminate interference from the hydrogen peroxide source, the electrode underwent continuous cyclic voltammetry scanning in four stages, each comprising eight complete cycles. Finally, the signals were acquired using electrochemical EIS and DPV.

### The effective working area of microelectrode

The reversible electron transfer process of electrochemical reaction involving free diffusion redox substances follows the Randles–Sevcik equation:

$${i}_{p}=\left(2.69 \times {10}^{5}\right){n}^{3/2}A{D}_{0}^{1/2}{\nu }^{1/2}{c}_{0}$$

$A$: electrode-active area;

${i}_{p}$: the redox peak current;

$n$: the number of electrons transferred in the redox reaction (for [Fe (CN)_6_]^3−/4−^,$n=1$);

${D}_{0}$: diffusion coefficient (for [Fe (CN)_6_]^3−/4−^, ${D}_{0}=6.7\times {10}^{-6} {cm}^{2} {s}^{-1}$, at 25 ℃);

$\nu$: the scan rate of the CV measurement (0.05 V);

${c}_{0}$: the concentration of the reactant (5 mM [Fe (CN)_6_]^3−/4−^). Note that 5 mM should be converted to $5\times {10}^{-6} mol/{cm}^{3}$.

The typical cyclic voltammograms of gold microelectrodes with a diameter of 200 μm used in this experiment in 5 mM [Fe (CN)_6_]^3−/4−^ were given in Additional file 1: Fig. S3b. The values of the anodic peak current and the cathodic peak current have been marked in the graph, and the mean value of them is ${i}_{p}=\frac{\left(ip\right)a+\left(ip\right)c}{2}=3.522\times {10}^{-7} A$.

By taking ${i}_{p}$ and other given parameters into the Randles–Sevcik equation, we can calculate effective working area of the microelectrode:

$$A=4.5242\times {10}^{-4} {cm}^{2}$$

Furthermore, the effective radius of the gold microelectrode is calculated as 120 μm. This is slightly larger than the radius of 100 μm marked by the specification. The possible reason is that there are some irregular areas extending outward around the Au microelectrode, as shown in Additional file 1: Fig. S2b.

### Data processing instructions

#### EIS data

According to the derivation of electrochemical impedance $Z\left(\omega \right)$ in the double layer model of electrode–electrolyte interface by Park et al [44]:

$$Z\left(\omega \right)={R}_{s}+\frac{{R}_{ct}}{1+{\omega }^{2}{R}_{ct}^{2}{C}_{d}^{2}}-\frac{j\omega {R}_{ct}^{2}{C}_{d}}{1+{\omega }^{2}{R}_{ct}^{2}{C}_{d}^{2}}=Z^{\prime}+jZ^{{\prime}{\prime}}$$

$$Z$$: impedance;

$$\omega$$: frequency;

${R}_{ct}$: charge transfer resistance;

$${R}_{s}$$: ohmic resistance;

${C}_{d}$: electric double layer capacitor.

For $\omega \to 0$, equation becomes $Z\left(\omega \right)={R}_{s}+{R}_{ct}$, which is an intercept on the $Z{\prime}\left(\omega \right)$ axis on the low-frequency side (charger transfer). Considering that ${R}_{ct}\gg {R}_{s}$ in this manuscript, the intercept of the low-frequency side (charge transfer) on the $Z^{\prime}\left(\omega \right)$ axis is approximately equal to ${R}_{ct}$. In the manuscript, it is expressed as $Z$ (impedance).

#### DPV data

The DPV signal is represented by its peak current value (Additional file 1: Fig. S4d). The peak current value can be automatically read out by CHI 660e software.

### Statistical analysis

All experiments and assays were repeated at least three times. The data were expressed as mean ± s.d. and compared by Student’s t test. The criteria used to estimate the limit of detection (LOD) is by measuring replicates of 10 blank samples and calculating the mean result and the standard deviation (LOD = mean of limit of blank + 3 × (standard deviation of blank sample). The Origin 9 were used for data analysis. All of the illustrations were generated using Adobe Illustrator CC 2019.

## Results and discussion

### Preparation and characterization of exosomes and Apt-TDNA

**Fig. 2:**
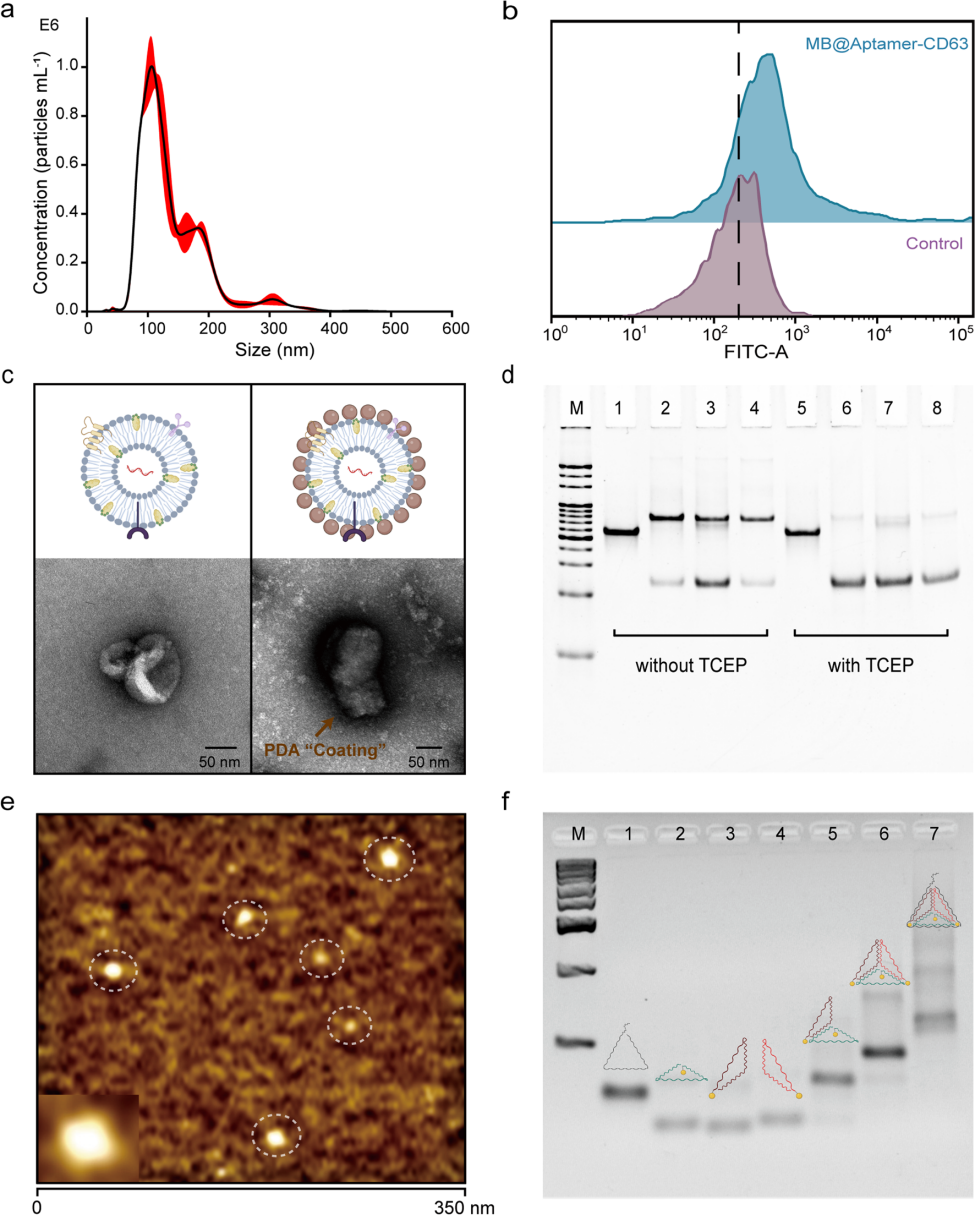
Characterization of exosomes and Apt-TDNA. **a** Characterization of size and number of exosomes using nanoparticle tracking analysis. **b** The widespread distribution of CD63 protein on the membrane of A549-derived exosomes was characterized by flow cytometry. **c** Purified bare exosomes (left) and PDA coated exosomes (right) observed using TEM. **d** Four Apt-TDNA monomers before (1–4) and after (5–8) TCEP pretreatment were characterized by 12.5% PAGE. **e** Characterization of Apt-TDNA by AFM. **f** In 2.5% agarose, the migration rates of thiolated monomers, assembly intermediate and Apt-TDNA are from fast to slow

Exosome enrichment and purification were carried out using ultracentrifugation and 0.22 μm filtration [45, 46]. The size distribution of A549-derived exosomes was determined using nanoparticle tracking analysis (NTA), with a peak at approximately 70–110 nm (Fig. 2a). Magnetic beads conjugated with CD63 aptamer were utilized to capture DiO-stained exosomes, and an increase in fluorescence was observed under flow cytometry compared to bare magnetic beads, confirming the widespread distribution of CD63 proteins on the membrane of A549-derived exosomes (Fig. 2b). As shown in Fig. 2c, the exosomes observed under transmission electron microscope (TEM) were manifested as cup-shaped vesicles with a diameter of approximately 100 nm. Furthermore, the morphology of exosomes deposited with PDA was characterized by TEM to demonstrate the feasibility of coating. After staining with uranyl acetate, a distinct rough and black film structure was observed on the surface of the coated exosomes, owing to the high electron density of the PDA film [32, 33] (Fig. 2d).

Before the assembly of Apt-TDNA, the efficacy of TCEP in dissociating disulfide bonds was investigated by exploring the molar ratio of TCEP to thiol-modified ssDNA and the working conditions of the solution (Additional file 1: Fig. S1). The molar ratio of TCEP to thiolated ssDNA as 1000:1 and 1.5 × PBS buffer were chosen. Channels 5–8 showed a significant decrease in the formation of unexpected disulfide bonds by the thiolated aptamers themselves after TCEP treatment (Fig. 2d). The morphology and formation process of Apt-TDNA were investigated by atomic force microscope (AFM) and 2.5% agarose gel electrophoresis, respectively. AFM image displayed the spatial structure of Apt-TDNA with a side length of about 5 nm (Fig. 2e). Gel analysis revealed the migration of four Apt-TDNA monomers (Lanes 1–4) and the generation of Apt-TDNA as the number of monomers increased (Lanes 5–7, Fig. 2f).

### Ingenious diamond-silica polishing system

Conventional polishing systems using aluminum oxide and suede are not suitable for microelectrodes ‘drawn’ from softened glass tubes, unlike polytetrafluoroethylene (PTFE)-wrapped electrodes. In our study, we discovered that the combination of a 0.01 µm grain size diamond polishing pad with a 75 nm silica polishing compound yielded excellent results for polishing this glass ‘drawn’ microelectrode (Additional file 1: Fig. S2). Before assembling Apt-TDNA on the electrode surface, a thorough cleaning procedure was performed, involving the use of piranha solution, ethanol, and deionized water, followed by careful polishing with a 75 nm silica polishing compound on a diamond polishing pad. These steps are crucial for the sensor’s performance and stability.

### Characterization of sensor assembly

**Fig. 3:**
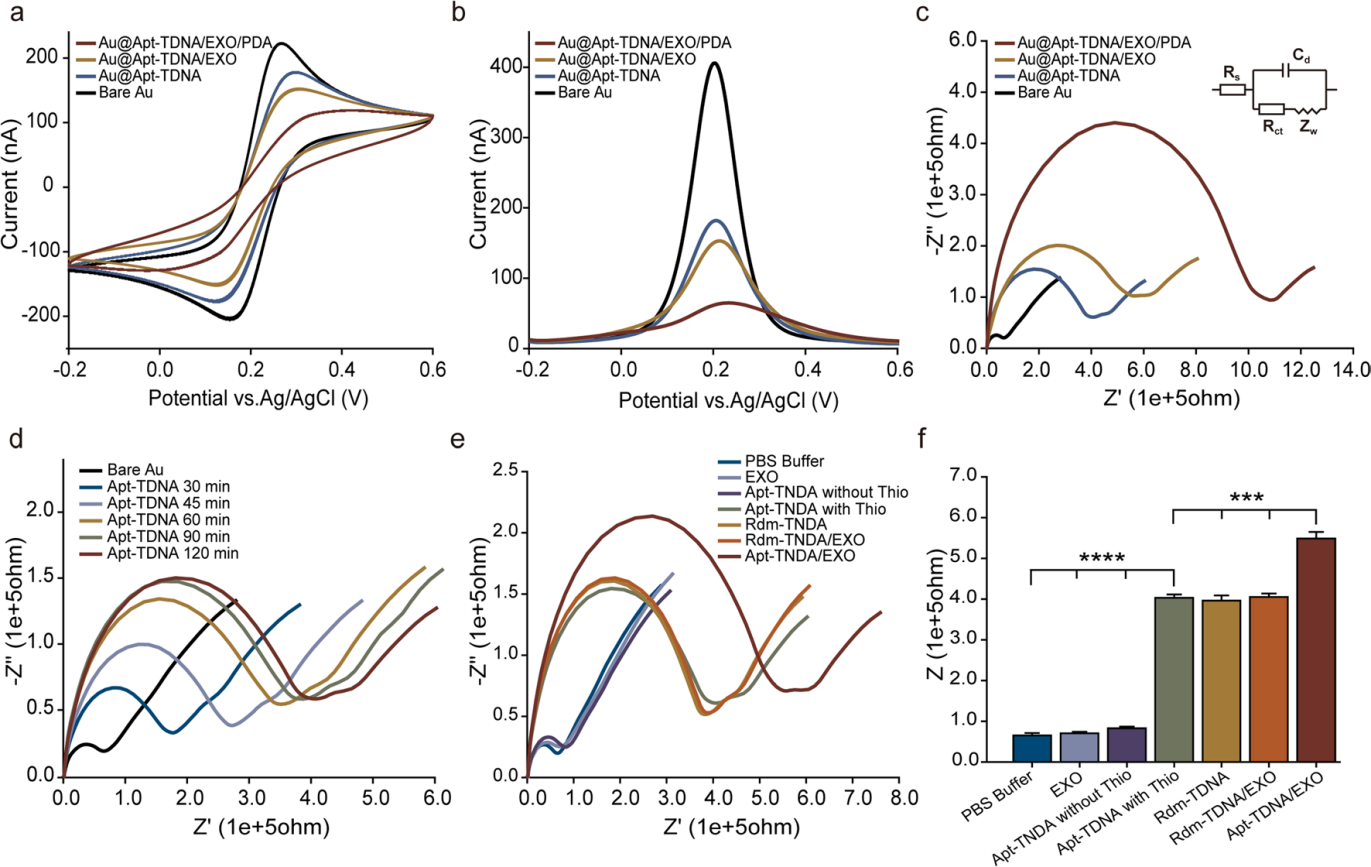
Electrochemical characterization of the construction process of a PDA-assisted Apt-TDNA microelectrode sensor for exosome detection. **a** CV. **b** DPV. **c** EIS. **d** Anchoring time optimization of Apt-TDNA. **e**, **f** Feasibility of Apt-TDNA microelectrode sensor and evaluation of non-specific binding. Data represent mean ± SD, n = 3, three technical replicates. Two-tailed Student’s t tests were used for comparisons, ***P < 0.001, ****P < 0.0001

After Apt-TDNA modification and MCH blocking treatment, the interface was allowed to ‘age’ in PBS buffer with 0.5 M NaCl to improve the stability and hybridization efficiency of the conjugates [47–49]. The purified and resuspended exosomes were incubated with the Apt-TDNA microelectrode and specifically captured. Subsequently, the whole electrode was immersed in freshly prepared PDA solution for the coating of PDA on the membrane of exosomes with a dense structure. From the self-assembly of Apt-TDNA on the electrode surface, the capture of exosomes to the PDA coating, each process increased the complexity of the electrode surface, hindering the path of charged ions to the electrode surface to varying degrees. Significant differences in signals of CV, DPV and EIS confirmed the formation of the sensor at each stage and showed the feasibility of the electrochemical sensor for exosome detection with the assistance of PDA (Fig. 3a–c).

It is essential to optimize the input parameters of the electrochemical workstation for sensitive detection of different reaction systems (Additional file 1: Fig. S4a–c). Unless otherwise noted, the parameters are set as follows: EIS (amplitude 10 mV, frequency 0.1 ~ 10^5^ Hz) and DPV (amplitude 50 mV, step size 2 mV). Compared with traditional gold electrodes and screen-printed electrodes, the current density flowing through the working microelectrode in this system is larger, the diffusion rate of substances on the electrode surface is faster, and the IR (Voltage)-drop is negligible [50, 51].

Meanwhile, the incubation time of Apt-TDNA with the microelectrode was also optimized. As shown in Fig. 3d, the impedance signal rapidly increased with increasing incubation time within the first hour, indicating continuous anchoring of Apt-TDNA to the microelectrode surface. After one hour, the impedance signal increased slowly and eventually stabilized, suggesting that the immobilization of Apt-TDNA on the microelectrode surface reached dynamic saturation. Thus, an incubation time of 120 min was selected for subsequent experiments. The well-defined orientation of Apt-TDNA on the electrode surface is the premise for the aptamer to maintain a fixed distance from the electrode surface and stretch normally to capture exosomes. Optimization of the incubation time for exosomes with the sensor, as shown in Additional file 1: Fig. S5, revealed that 90 min ensured capture of the vast majority of exosomes.

In Fig. 3e, f, compared to the nonspecific adsorption of a bare Au microelectrode in PBS solution, exosome solution, and non-thiolated Apt-TDNA (Apt-TDNA w/o thio) solution, only the thiol-modified Apt-TDNA (Apt-TDNA with thio) successfully anchored onto the electrode surface through the Au–S interaction, resulting in a distinct impedance signal. Moreover, we employed a 32-nt random ssDNA sequence (Tha2, Additional file 1: Table S1) involved in the assembly of Rdm-TDNA (negative control). In contrast, only the Apt-TDNA sensor exhibited a significant increase in signal.

### Quantification of exosomes by Apt-TDNA microelectrode sensor

**Fig. 4:**
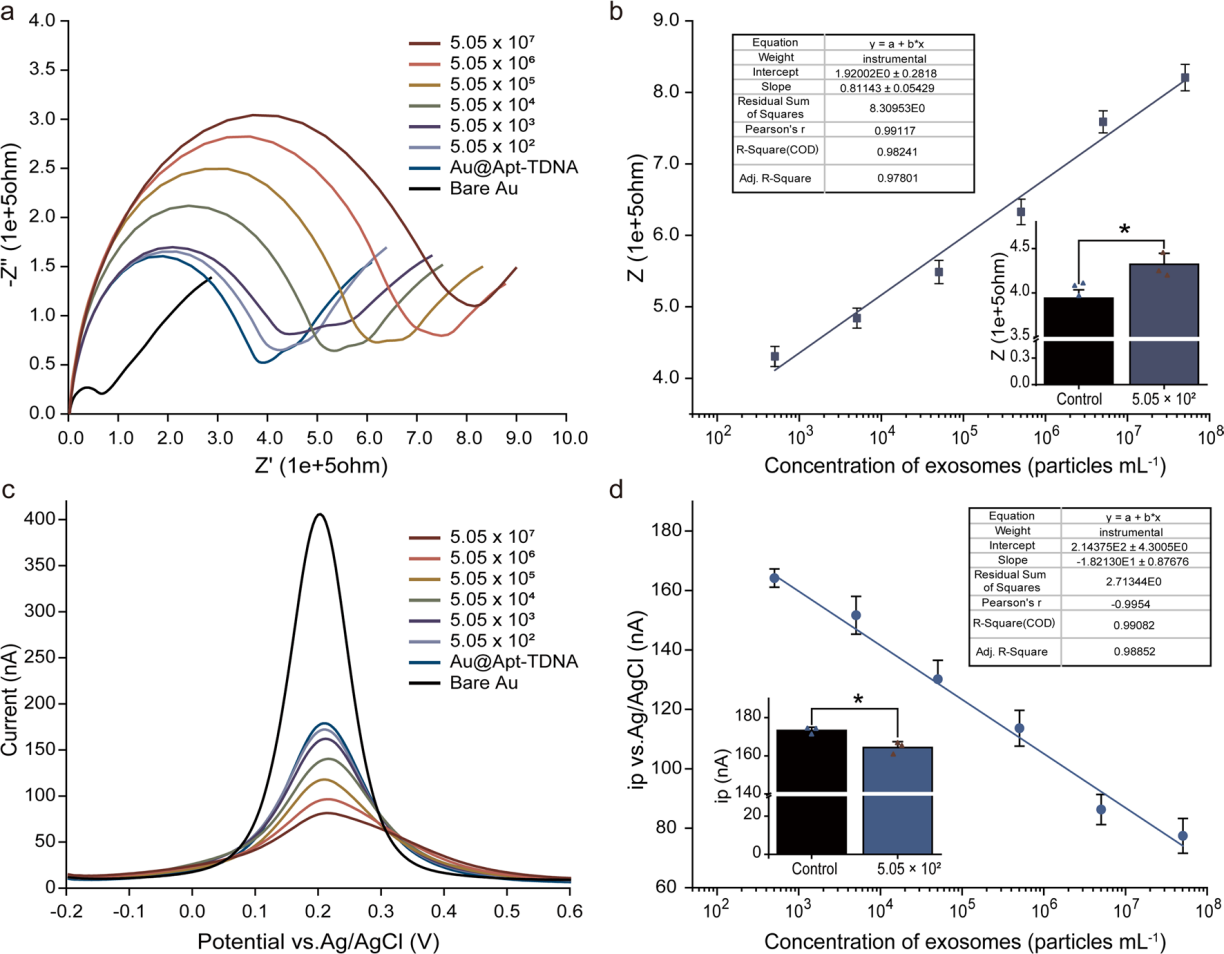
The detection of purified cancerous exosomes with Apt-TDNA microelectrode sensor. **a** The typical impedance intensity curves in the presence of varying exosome levels. **b** Calibration curve of exosome concentration gradient versus Z. **c** Detection by DPV. **d** Calibration curve between peak current and exosome concentration. Data represent mean ± SD, n = 3, three technical replicates. Two-tailed Student’s t tests were used for comparisons, *P < 0.05

To evaluate the capability of the Apt-TDNA microelectrode sensor for quantifying target exosomes, EIS and DPV techniques were employed. DPV provides an overview of the entire system’s current state, while EIS focuses on the complexity of the surface of working electrode. In Fig. 4a, we present the background signal without exosomes and typical impedance intensity curves in the presence of varying exosome levels. We then calibrated the system against exosome concentration by measuring impedance ($Z$) responses (Fig. 4b). The Apt-TDNA microelectrode sensor successfully detected exosome as low as 6.92 × 10^2^ particles mL^−1^ using EIS, with a dynamic range up to 5.05 × 10^7^ particles mL^−1^. The linear equation after fitting is $y=1.92002x+0.81143$ (${R}^{2}=0.97801$). Furthermore, in Fig. 4c, d, we present the current responses of exosomes with varied concentrations using the DPV assay. A calibration curve was plotted relating exosome concentration to peak current (ip), with a limit of detection (LOD) of 4.15 × 10^2^ particles mL^−1^ and a dynamic range up to 5.05 × 10^7^ particles mL^−1^. The linear equation after fitting is $y=-1.82130{\text{E}}1x+2.14375{\text{E}}2$ (${R}^{2}=0.98852$).

### Ultrasensitive quantification of exosomes assisted by PDA signal amplification strategy

**Fig. 5:**
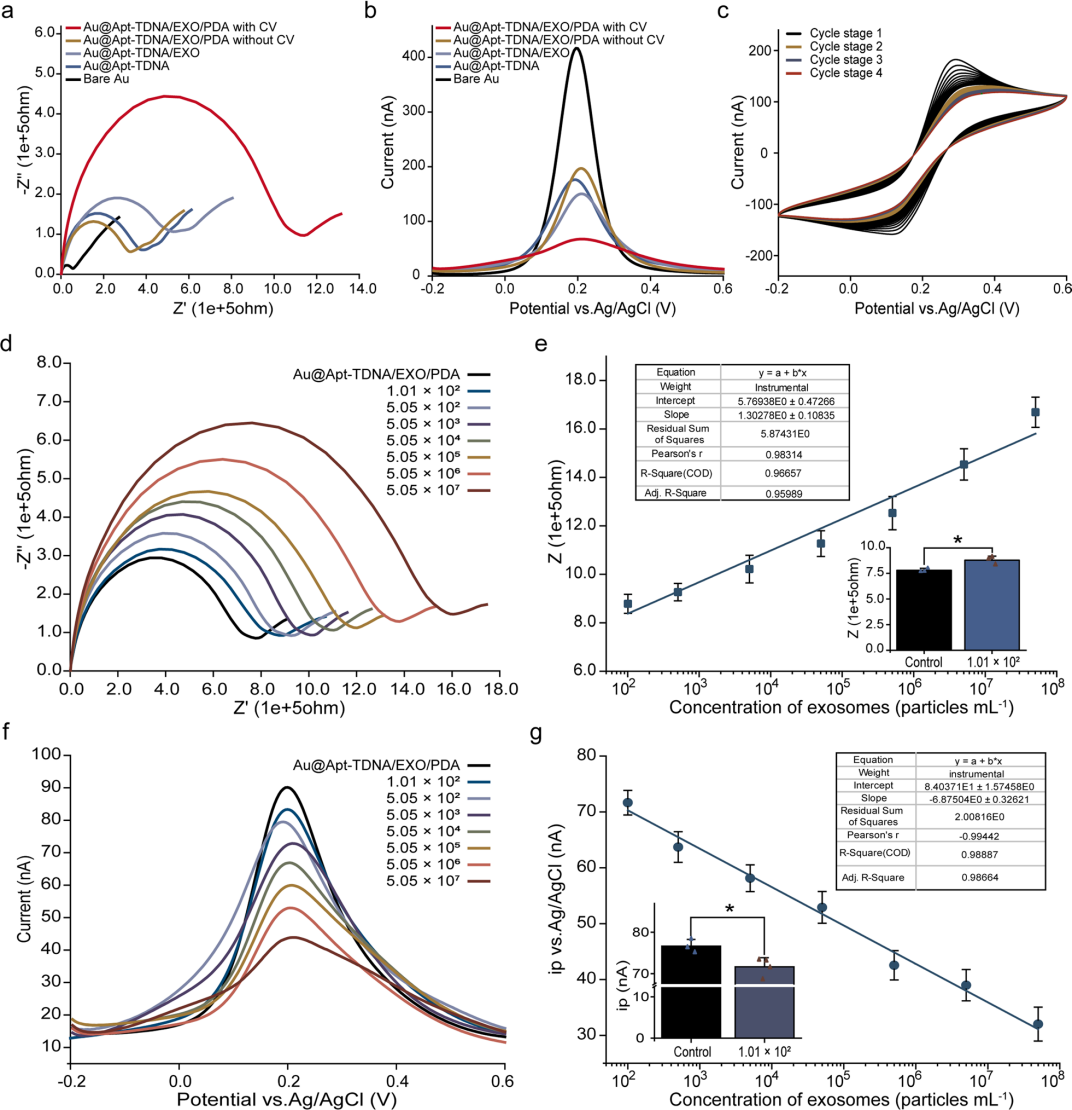
Detection of exosomes based on a signal amplification strategy assisted by PDA coating. Additional CV electrochemical deposition helps the true signal of PDA coating appear: (**a**) EIS, (**b**) DPV. (**c**) Continuous CV promotes the electrochemical deposition of PDA. **d** Typical impedance intensity curves in the presence of varying exosome levels. **e** Calibration curve of EIS. **f** Detection by DPV. **g** Calibration curve of DPV. Data represent mean ± SD, n = 3, three technical replicates. Two-tailed Student’s t tests were used for comparisons, *P < 0.05

The uniqueness of our work lies in depositing PDA on the membrane of exosomes captured by the Apt-TDNA microelectrode, which effectively enhances the complexity of the electrode surface and improves sensor sensitivity. Before the PDA coating, we optimized the amount of HRP needed for the catalytic oxidation of DA to PDA in homogeneous experiments (Additional file 1: Fig. S6). However, during the introduction of PDA into the reaction system, we observed a decrease in signal instead of the expected enhancement (Fig. 5a, b). We speculated that during PDA deposition, a small amount of H_2_O_2_ was trapped on the electrode surface, which could not be removed by simple rinsing. Additionally, a portion of the PDA remained in the intermediate state between quinone and phenol [52]. To enhance the formation of a dense and uniform coating on the electrode surface, we employed an electrochemical deposition method. A continuous CV scan was performed on the PDA-coated microelectrode, with each cycle stage consisting of 16 half cycles, until the CV curve reached a steady state (Fig. 5c). The resulting red curves in Fig. 5a, b represent the measured signal derived from PDA. Subsequently, we investigated the deposition rate of PDA by varying the coating time and observed that the deposition rate initially increased and then decreased over time (Additional file 1: Fig. S7). To strike a balance between duration and effectiveness, we selected a coating time of 90 min.

To further demonstrate the effectiveness of our unique PDA coating strategy for quantifying low concentration levels of exosomes, we conducted an experiment using an Apt-TDNA microelectrode incubated with different concentrations of exosomes. The microelectrodes were then coated with a PDA solution, and the resulting signal responses were recorded using EIS and DPV techniques, respectively. We plotted and calibrated the characteristic curves of the EIS (Fig. 5d, e) and DPV (Fig. 5f, g) signals for microelectrodes in the presence of varying amounts of exosomes with PDA coating. Importantly, the detection limits of both techniques were significantly improved, reaching as low as 1.39 × 10^2^ particles mL^−1^ (~ 14 exosomes) (EIS) and 6.27 × 10^1^ particles mL^−1^ (~ 6 exosomes) (DPV) with a dynamic range up to 5.05 × 10^7^ particles mL^−1^, respectively. The fitted linear equations are $y=1.30278x+5.76938$ (${R}^{2}=0.95989$) (EIS) and $y=-6.87504x+8.40371{\text{E}}1$ (${R}^{2}=0.98664$) (DPV), respectively. The introduction of PDA coating approach resulted in an impressive increase in the sensitivity of exosome detection, surpassing that of Apt-TDNA microelectrode alone by more than threefold.

### Stability of the Apt-TDNA microelectrode sensor

Additionally, the storage stability of Apt-TDNA microelectrode sensor was investigated (Additional file 1: Fig. S8). The results show that when the Apt-TDNA microelectrode was stored for 3 and 7 days in N_2_ gas at 4 ℃, the Z signal intensity after PDA coating was maintained at 91.7% and 81.9% of freshly made, respectively. The possible reason for the decrease in signal is attributed to the degradation of Apt-TDNA.

### Comparison of different exosome detection methods

As shown in Table 1, the PDA-assisted Apt-TDNA microelectrode sensor has a lower detection limit compared to other sensors used for exosome detection. Additionally, it enables simultaneous EIS and DPV dual detection. We anticipate that the PDA-assisted Apt-TDNA microelectrode sensor could play an active role in early cancer diagnosis.

**Table 1:** Comparison of different exosome detection methods

Method	Strategy	Dynamic range (particles mL^−1^)	LOD (particles mL^−1^)	Refs.
SPR	Dual gold nanoparticle-assisted signal amplification	–	5.0 × 10^3^	[11]
FCM	Rolling circle amplification-assisted flow cytometry	1.0 × 10^5^–1.0 × 10^10^	1.32 × 10^5^	[53]
SERS	Colocalization-dependent system and ratiometric SERS sensing based on gold nanorod arrays	1.0 × 10^4^–5.0 × 10^6^	5.3 × 10^3^	[54]
Fluorescence	Aptamer-initiated catalytic hairpin assembly fluorescence assay	8.4 × 10^3^–8.4 × 10^8^	5.0 × 10^2^	[55]
Colorimetry	Aptamer-conjugated horseradish peroxidase catalyzed polydopamine coloration	1.0 × 10^3^–1.0 × 10^11^	7.77 × 10^3^	[29]
ECL	Ti3C2Tx − Bi2S3 − x eterostructure/engineered lipid layer based nanoarchitecture	6.0 × 10^4^–1.0 × 10^8^	3.6 × 10^4^	[56]
Electrochemistry	Ratiometric biosensor based on MB/Fc	1.0 × 10^5^–1.0 × 10^11^	1.51 × 10^4^	[57]
Electrochemistry	Hierarchical nanostructuring array and primer exchange reaction	1.4 × 10^2^–1.4 × 10^7^	7.5 × 10^1^	[58]
Electrochemistry	Ultra-thin covalent organic framework nanosheets coupled with CRISPR-Cas12a mediated signal amplification	1.2 × 10^5^–1.2 × 10^10^	3.8 × 10^4^	[59]
Electrochemistry	Peptide-templated AgNPs nanoprobe	1.0 × 10^5^–1.0 × 10^11^	3.7 × 10^4^	[60]
Electrochemistry	PDA-assisted Apt-TDNA microelectrode sensor	1.39 × 10^2^–5.05 × 10^7^	6.27 × 10^1^	This work

Surface plasmon resonance (*SPR*), Flow Cytometry (*FCM*), Surface-enhanced Raman spectroscopy (*SERS*), Electrochemiluminescence (*ECL*)

## Conclusions

In summary, we present a novel Apt-TDNA microelectrode sensor for the simple, rapid, and sensitive electrochemical detection of cancerous exosomes, aided by a PDA coating signal amplification strategy. The precise immobilization of Apt-TDNA ensures extension of aptamers in the liquid phase and efficient exosome capture. Before PDA coating, the sensor only involves a 90 min exosome capture process, achieving a limit of detection of 6.92 × 10^2^ particles mL^−1^ (EIS) and 4.15 × 10^2^ particles mL^−1^ (DPV). The PDA coating acts as a blocker for charged particles, further complementing the sensitive electrical signal feedback of the microelectrode. After PDA coating, the LOD of our sensor could be drastically reduced to 1.39 × 10^2^ particles mL^−1^ (~ 14 exosomes) (EIS) and 6.27 × 10^1^ particles mL^−1^ (~ 6 exosomes) (DPV) within an overall process time of 180 min. This approach simplifies the detection process and enhances the sensitivity of exosome detection to the single particle level. Our findings provide constructive insights for the diagnosis of cancer at early stage and further advancements in exosome-based diagnostic strategies.

The conventional molecular biology techniques for analyzing membrane proteins of exosomes in clinical application, consisting of western blot, enzyme-linked immunosorbent assay (ELISA), flow cytometry, as well as mass spectrometry, either involve multiple steps including protein isolation, fluorescent labeling, and data analysis or are time-consuming and relying on bulky and expensive equipment (Table 2). In contrast, our PDA-assisted Apt-TDNA microelectrode sensor has several advantages, for example, the sensor can capture and quantify exosomes in situ with sensitivity at the single-molecule level. In addition, it provides multiparameter analysis of exosomes without damaging their internal contents at low cost. Moreover, the sensor detects exosomes with the advantage of easy response control and point-of-care testing. However, the limitation of this work is that the sensor only targets one biomarker, which may not have high specificity in the detection of clinical samples. We are considering to improve the specificity of the sensor by targeting dual or more biomarkers. The improvement strategy could be as follows: After the sensor captures exosomes, another biotin-labeled aptamer targeting different membrane protein and streptavidin-labeled HRP are sequentially introduced in the system to form a sandwich structure, like ELISA. The dual recognition will improve the recognition specificity of the sensor and broaden its application in clinical diagnostics. Furthermore, the process of HRP catalyzing DA to PDA is correlated with the concentration of exosomes, which will further improve the sensitivity of the sensor.

**Table 2:** Comparison with conventional molecular biology techniques

Method	Detected objects	Mechanism	Advantages	Limitations	Refs.
ELISA	Proteins	Target exosome is adsorbed on the solid phase surface and recognized by enzyme-coupled antibodies	High reproducibility; reliability	Nonspecific adsorption of biomolecules	[61]
FCM	Proteins	Exosomes flow through a laser beam, where the light scattered is characteristic to the Exosomes	High-throughput, well established method	Relatively high cost for instrument	[62]
MS	Proteins	Separating the components of EVs by their mass and electrical charge	High-throughput and sensitivity	Relatively high cost for instrument; requires high purity of EVs	[63]
PDA-assisted Apt-TDNA microelectrode sensor	Proteins	Exosome capture and electron transfer between electroactive molecules and microelectrode surface	easy operation; fast analysis; low cost; high sensitivity	Specificity needs to be improved	This work

Enzyme-linked immunosorbent assay (*ELISA*), Flow Cytometry (*FCM*), Mass spectrometry (*MS*)

### Supplementary Information

**Additional file 1: Fig S1.** Optimization of TCEP treatment of thiol groups. **(a)** The band brightness of the disulfide product after incubation of TCEP and thiol-modified ssDNA for 2 h was observed under 12.5% PAGE. Molar excess ratio of TCEP relative to ssDNA (a: 0; b: 100; c: 300; d: 500; e: 1000; f: 3000; g: 5000; h:10000). **(b)** Under the treatment of TCEP equivalent to the 1000-fold molar equivalent of thiol-monomer, the effect of eliminating disulfide products under different concentrations of PBS buffer was investigated. Lanes 1-7 are as follows: ddH2O, 0.5× PBS, 1×, 1.5×, 2×, 5×, 8×). **Fig S2.** Morphology of the microelectrode surface magnified 400-fold under different polishing methods. **(a)** Tradition: polished with 1.0 µm, 0.3 µm, and 0.05 µm silica polishing powder on the brown suede velvet polishing cloth in turn for 4 min.** (b) **Innovation: polished with the interaction of 0.01 µm grain size diamond polishing pad and 75 nm silica polishing compound for 5 min. **Fig S3.** Electrochemical polishing and characterization of gold microelectrode. **(a)** The typical electrochemical polishing curve of microelectrode in 0.5 M sulphuric acid. **(b)** Typical CV (50 mV scan rate) of microelectrode in electrolyte buffer (5 mM Fe [(CN)6]3-/4-, 0.1 M KCl). **Fig S4.** Optimization of detection parameters of CHI 660e electrochemical workstation. **(a)** Amplitude optimization for DPV. **(b)** Step size optimization for DPV. **(c) **Amplitude optimization for EIS. **(d)** DPV peak current diagram. **Fig S5.** **(a)**,** (b)**: Optimization of incubation time of exosomes. Data represent mean ± SD, n = 3, three technical replicates. **Fig S6.** 900 nM HRP loading volume (µL) optimized. **Fig S7. (a)**,** (b)**: Optimization of coating time in PDA solution. Data represent mean ± SD, n = 3, three technical replicates. **Fig S8.** Stability test of the Apt-TDNA microelectrode sensor. **(a)** Typical impedance intensity curves of sensors with different storage time in the presence of exosomes (5.05 × 107 particles mL-1 ).** (b)** Signal recovery of sensors after different storage days. The Z value on the 0 day was set to 100%. **Table S1.** List of the DNA sequences used in this work

## Data Availability

All data generated or analyzed during this study are included in this published article.

## Abbreviations

Apt-TDNA: Aptamer-carrying nano-tetrahedral DNA
PDA: Polydopamine
DA: Dopamine
HRP: Horseradish peroxidase
EIS: Electrochemical impedance spectroscopy
DPV: Differential pulse voltammetry
RC: Resistance–capacitance
PCR: Polymerase chain reaction
APTES: 3-Triethoxysilylpropylamine
CV: Cyclic voltammetry
MCH: 6-Mercaptohexanol
NTA: Nanoparticle tracking analysis
TEM: Transmission electron microscopy
TCEP: Tris(2-carboxyethyl)phosphine hydrochloride
AFM: Atomic force microscopy
PTFE: Polytetrafluoroethylene
Z: Impedance
ip: Peak current
LOD: Limit of detection
SPR: Surface plasmon resonance
FCM: Flow cytometry
SERS: Surface-enhanced Raman spectroscopy
ECL: Electrochemiluminescence
ELISA: Enzyme-linked immunosorbent assay
MS: Mass spectrometry
