# Correction: Polydopamine-assisted aptamer-carrying tetrahedral DNA microelectrode sensor for ultrasensitive electrochemical detection of exosomes

**DOI:** 10.1186/s12951-024-02347-1

**Published:** 2024-02-28

**Authors:** Bowen Jiang, Tenghua Zhang, Silan Liu, Yan Sheng, Jiaming Hu

**Affiliations:** 1 International Joint Laboratory of Catalytic Chemistry, State Key Laboratory of Advanced Special Steel, Innovation Institute of Carbon Neutrality, College of Sciences, Shanghai University, Shanghai, 200444 China; 2 Institute of Translational Medicine, Shanghai University, Shanghai, 200444 China; 3 MOE Key Laboratory of Laser Life Science & Institute of Laser Life Science, Guangdong Provincial Key Laboratory of Laser Life Science, College of Biophotonics, South China Normal University, Guangzhou, 510631 China

**Correction: Journal of Nanobiotechnology (2024)22:55**

10.1186/s12951-024-02318-6

Following publication of the original article, details for affiliations of all authors were incorrectly given as:

1 MOE Key Laboratory of Laser Life Science & Institute of Laser Life Science, Guangdong Provincial Key Laboratory of Laser Life Science, College of Biophotonics, South China Normal University, Guangzhou 510631, China.

2 Institute of Translational Medicine, Shanghai University, Shanghai 200444, China.

3 International Joint Laboratory of Catalytic Chemistry, State Key Laboratory of Advanced Special Steel, Innovation Institute of Carbon Neutrality, College of Sciences, Shanghai University, Shanghai 200444, China.

The correct affiliations should have been:

1 International Joint Laboratory of Catalytic Chemistry, State Key Laboratory of Advanced Special Steel, Innovation Institute of Carbon Neutrality, College of Sciences, Shanghai University, Shanghai, 200444, China.

2 Institute of Translational Medicine, Shanghai University, Shanghai, 200444, China.

3 MOE Key Laboratory of Laser Life Science & Institute of Laser Life Science, Guangdong Provincial Key Laboratory of Laser Life Science, College of Biophotonics, South China Normal University, Guangzhou, 510631, China.

The original article has been corrected.

