# Supplementary material for: Polydopamine-assisted aptamer-carrying tetrahedral DNA microelectrode sensor for ultrasensitive electrochemical detection of exosomes
Source: J Nanobiotechnology. 2024 Feb 8;22:55. doi: 10.1186/s12951-024-02318-6 (PMC10854160; doi:10.1186/s12951-024-02318-6)
Supplement: Supplementary file 1 — Additional file 1: Fig S1. Optimization of TCEP treatment of thiol groups. (a) The band brightness of the disulfide product after incubation of TCEP and thiol-modified ssDNA for 2 h was observed under 12.5% PAGE. Molar excess ratio of TCEP relative to ssDNA (a: 0; b: 100; c: 300; d: 500; e: 1000; f: 3000; g: 5000; h:10000). (b) Under the treatment of TCEP equivalent to the 1000-fold molar equivalent of thiol-monomer, the effect of eliminating disulfide products under different concentrations of PBS buffer was investigated. Lanes 1-7 are as follows: ddH2O, 0.5× PBS, 1×, 1.5×, 2×, 5×, 8×). Fig S2. Morphology of the microelectrode surface magnified 400-fold under different polishing methods. (a) Tradition: polished with 1.0 µm, 0.3 µm, and 0.05 µm silica polishing powder on the brown suede velvet polishing cloth in turn for 4 min. (b) Innovation: polished with the interaction of 0.01 µm grain size diamond polishing pad and 75 nm silica polishing compound for 5 min. Fig S3. Electrochemical polishing and characterization of gold microelectrode. (a) The typical electrochemical polishing curve of microelectrode in 0.5 M sulphuric acid. (b) Typical CV (50 mV scan rate) of microelectrode in electrolyte buffer (5 mM Fe [(CN)6]3-/4-, 0.1 M KCl). Fig S4. Optimization of detection parameters of CHI 660e electrochemical workstation. (a) Amplitude optimization for DPV. (b) Step size optimization for DPV. (c) Amplitude optimization for EIS. (d) DPV peak current diagram. Fig S5. (a), (b): Optimization of incubation time of exosomes. Data represent mean ± SD, n = 3, three technical replicates. Fig S6. 900 nM HRP loading volume (µL) optimized. Fig S7. (a), (b): Optimization of coating time in PDA solution. Data represent mean ± SD, n = 3, three technical replicates. Fig S8. Stability test of the Apt-TDNA microelectrode sensor. (a) Typical impedance intensity curves of sensors with different storage time in the presence of exosomes (5.05 × 10^7^ particles mL-1 ). (b) Signal recovery of s [file 12951_2024_2318_MOESM1_ESM.docx]

**Additional file**

**Polydopamine-assisted Aptamer-carrying Tetrahedral DNA Microelectrode Sensor for Ultrasensitive Electrochemical Detection of Exosomes**

Bowen Jiang^c^, Tenghua Zhang^c^, Silan Liu^c^, Yan Sheng^b,c,^*, Jiaming Hu^a,c,^**

^a^ International Joint Laboratory of Catalytic Chemistry, State Key Laboratory of Advanced Special Steel, Innovation Institute of Carbon Neutrality, College of Sciences, Shanghai University, Shanghai 200444, China

^b^ Institute of Translational Medicine, Shanghai University, Shanghai 200444, China.

^c^ MOE Key Laboratory of Laser Life Science & Institute of Laser Life Science, Guangdong Provincial Key Laboratory of Laser Life Science, College of Biophotonics, South China Normal University, Guangzhou 510631, China.

* Corresponding author at: Institute of Translational Medicine, Shanghai University, Shanghai 200444, China.

** Corresponding author at: International Joint Laboratory of Catalytic Chemistry, State Key Laboratory of Advanced Special Steel, Innovation Institute of Carbon Neutrality, College of Sciences, Shanghai University, Shanghai 200444, China.

E-mail addresses: [ysheng@shu.edu.cn](mailto:ysheng@m.scnu.edu.cn) (Y. Sheng), [jmhu@shu.edu.cn](mailto:jmhu@m.scnu.edu.cn) (J. Hu)

**Supplementary figures**

**S1. Optimization of TCEP treatment of thiol groups.**

**
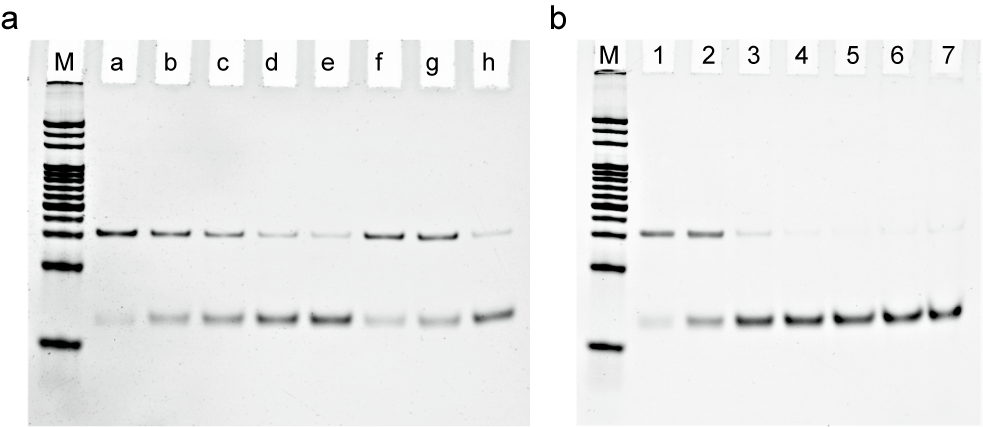
**

**Fig. S1** Optimization of TCEP treatment of thiol groups. (a) The band brightness of the disulfide product after incubation of TCEP and thiol-modified ssDNA for 2 h was observed under 12.5% PAGE. Molar excess ratio of TCEP relative to ssDNA (a: 0; b: 100; c: 300; d: 500; e: 1000; f: 3000; g: 5000; h:10000). (b) Under the treatment of TCEP equivalent to the 1000-fold molar equivalent of thiol-monomer, the effect of eliminating disulfide products under different concentrations of PBS buffer was investigated. Lanes 1-7 are as follows: ddH_2_O, 0.5× PBS, 1×, 1.5×, 2×, 5×, 8×).

**S2. The flatness of microelectrode surface under different polishing methods.**

**
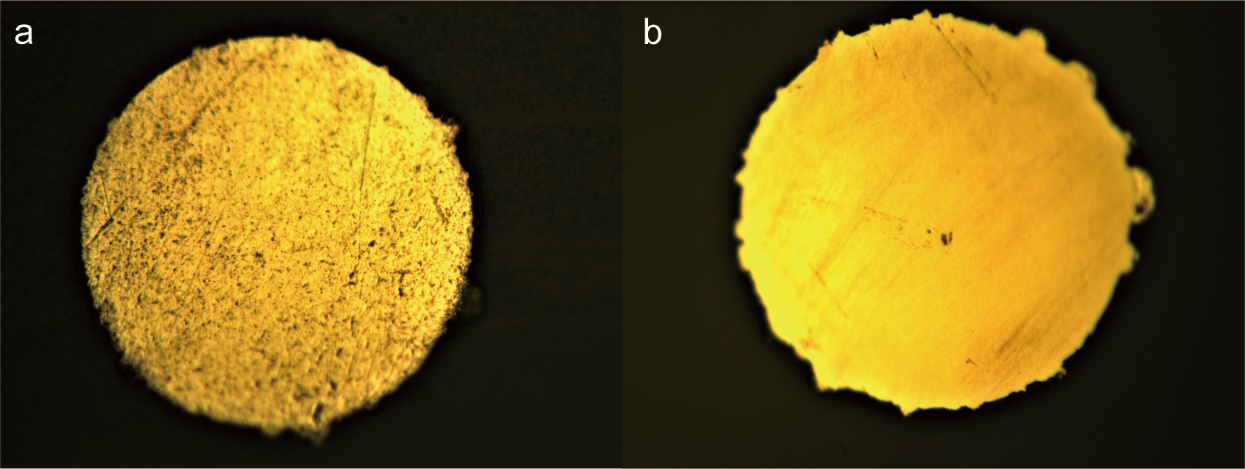
**

**Fig. S2** Morphology of the microelectrode surface magnified 400-fold under different polishing methods. (a) Tradition: polished with 1.0 µm, 0.3 µm, and 0.05 µm silica polishing powder on the brown suede velvet polishing cloth in turn for 4 min. (b) Innovation: polished with the interaction of 0.01 µm grain size diamond polishing pad and 75 nm silica polishing compound for 5 min.

**S3. Electrochemical polishing and characterization of gold microelectrode.**

**
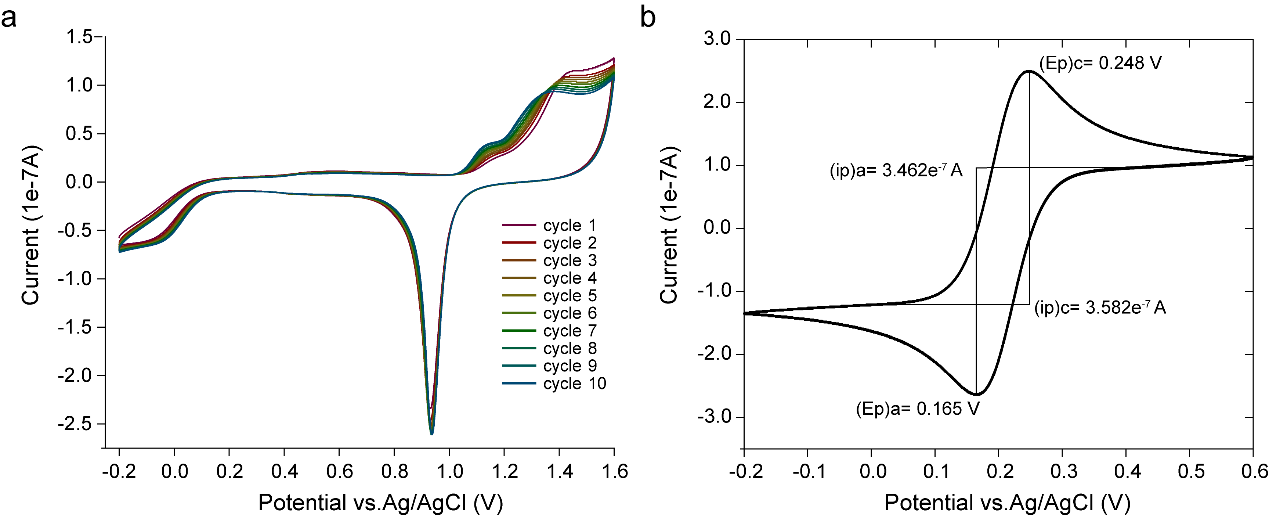
**

**Fig. S3** Electrochemical polishing and characterization of gold microelectrode. (a) The typical electrochemical polishing curve of microelectrode in 0.5 M sulphuric acid. (b) Typical CV (50 mV scan rate) of microelectrode in electrolyte buffer (5 mM Fe [(CN)_6_]^3-/4-^, 0.1 M KCl).

**S4. Optimization of input parameters for EIS and DPV.**

**
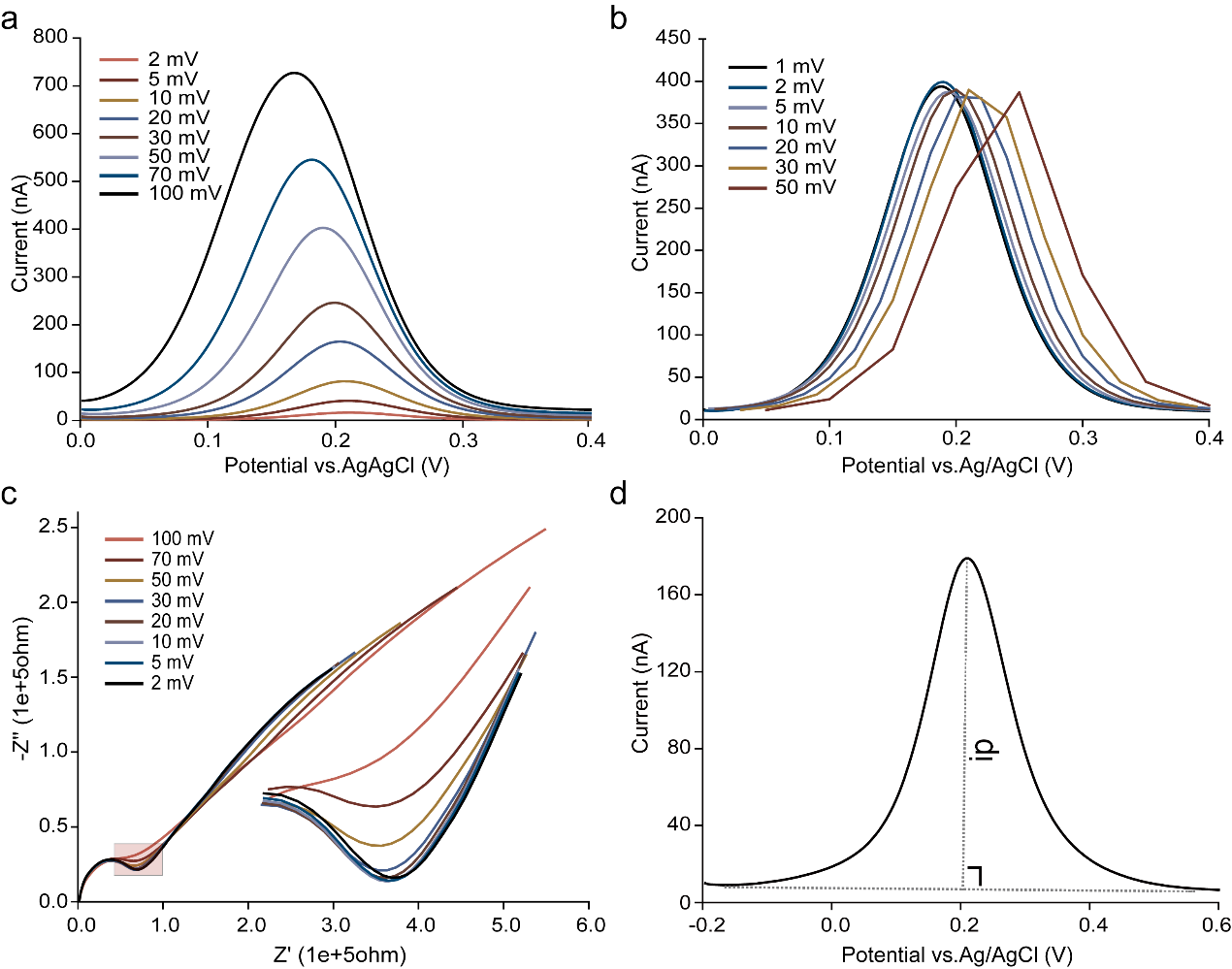
**

**Fig. S4** Optimization of detection parameters of CHI 660e electrochemical workstation. (a) Amplitude optimization for DPV. (b) Step size optimization for DPV. (c) Amplitude optimization for EIS. (d) DPV peak current diagram.

**S5. Optimization of incubation time of exosomes.**

**
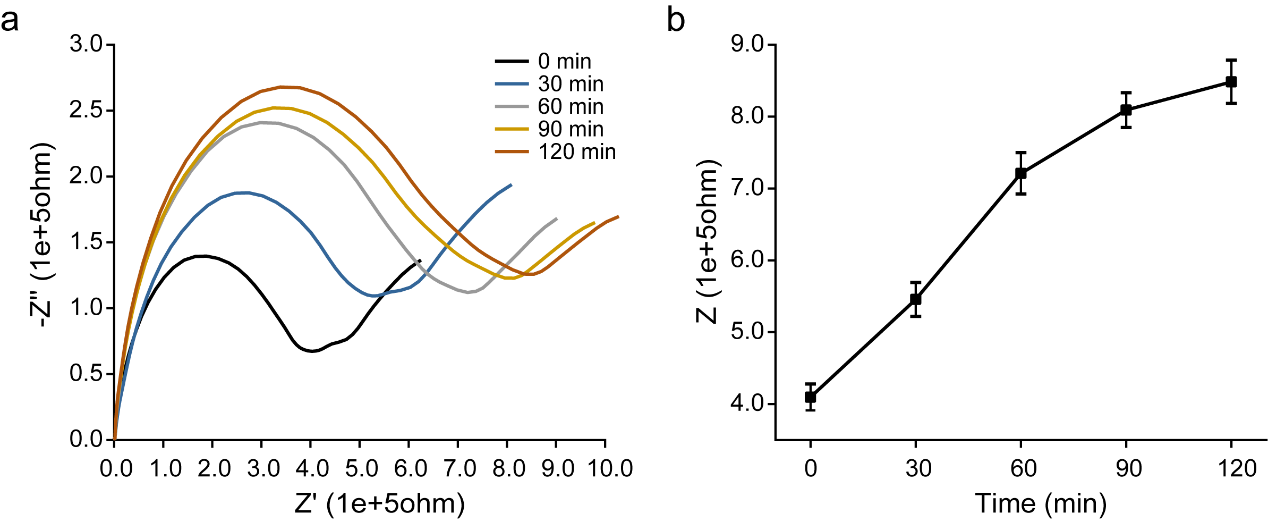
**

**Fig. S5** (a), (b): Optimization of incubation time of exosomes. Data represent mean ± SD, n = 3, three technical replicates.

**S6. Optimization of HRP for catalytic oxidation of DA to PDA.**

**
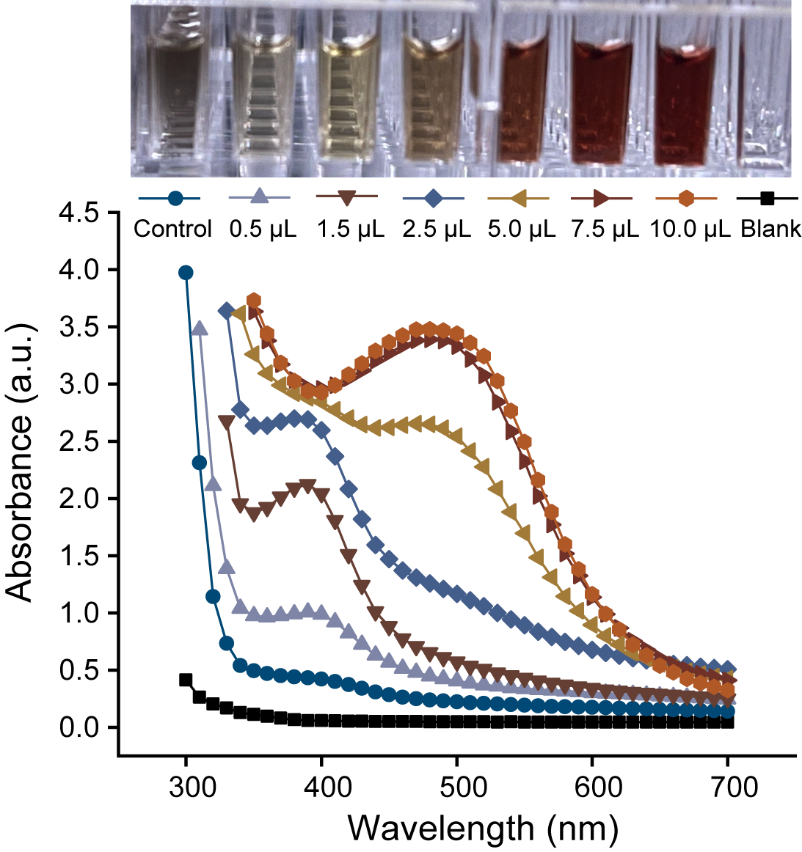
**

**Fig. S6** 900 nM HRP loading volume (µL) optimized.

**S7. Optimization of coating time in PDA solution.**

**
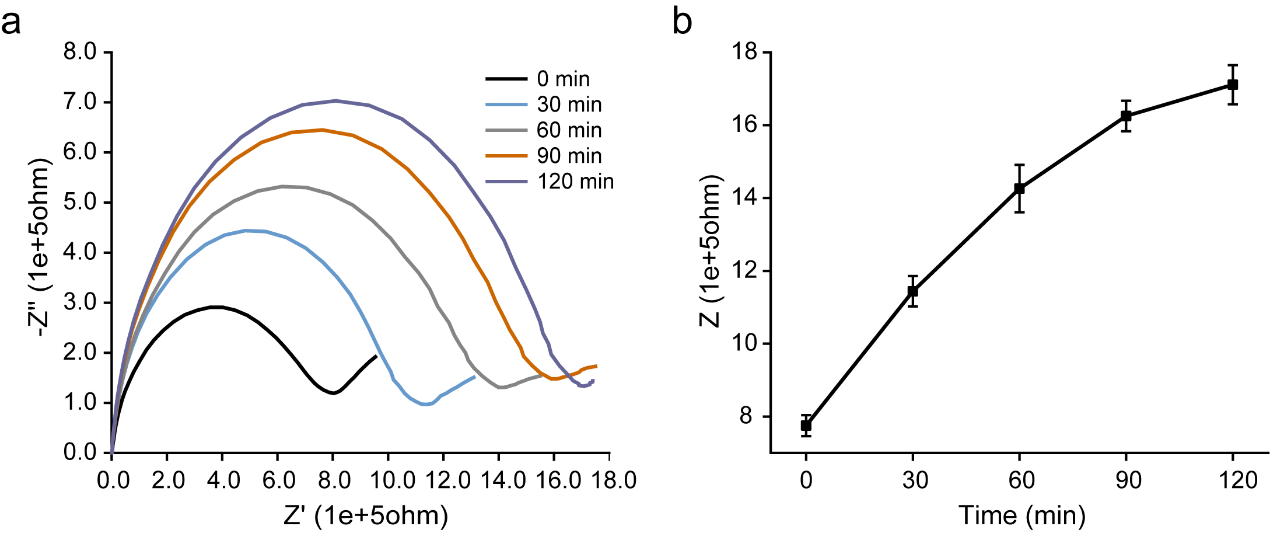
**

**Fig. S7** (a), (b): Optimization of coating time in PDA solution. Data represent mean ± SD, n = 3, three technical replicates.

**S8. Stability test of the Apt-TDNA microelectrode sensor.**

**
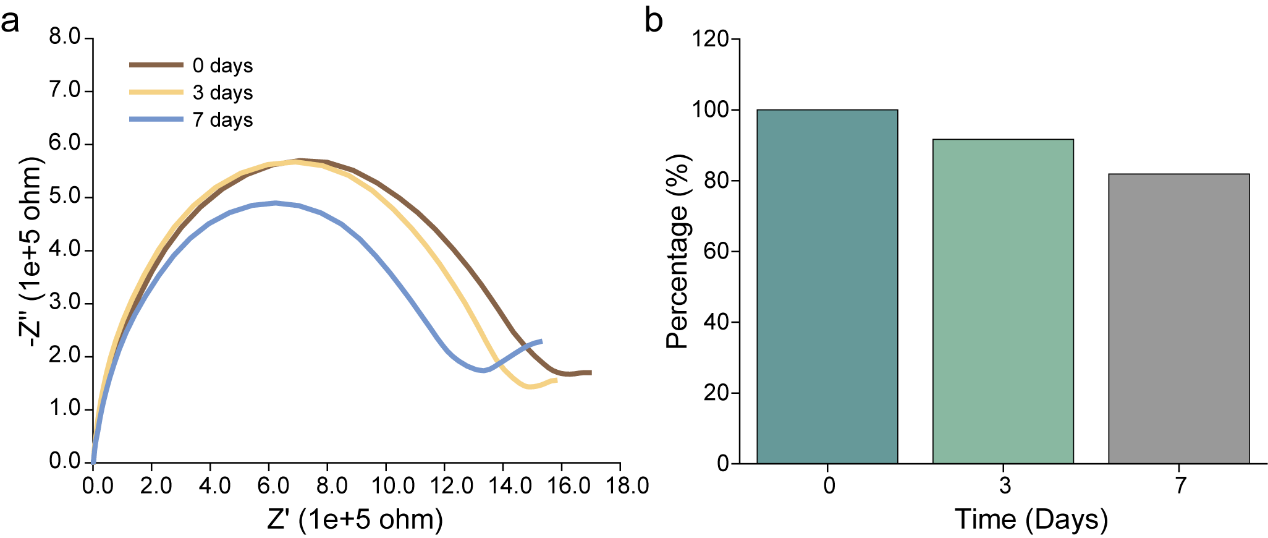
**

**Fig. S8** Stability test of the Apt-TDNA microelectrode sensor. (a) Typical impedance intensity curves of sensors with different storage time in the presence of exosomes (5.05 × 10^7^ particles mL^-1^ ). (b) Signal recovery of sensors after different storage days. The Z value on the 0 day was set to 100%.

**Additional file table**

**Table S1. List of the DNA sequences used in this work**

**
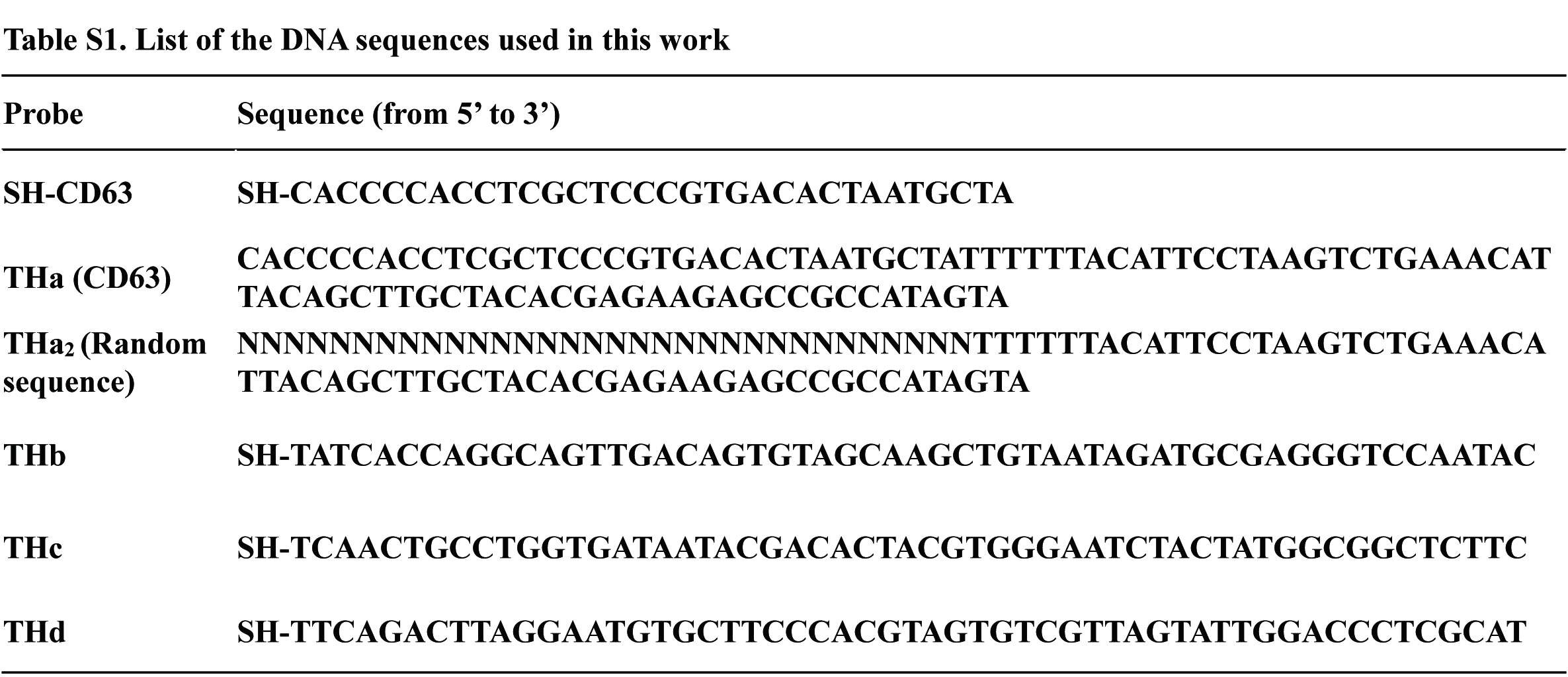
**
